# Integrity of plasma DNA is inversely correlated with vaccine-induced antitumor immunity in ovarian cancer patients

**DOI:** 10.1007/s00262-020-02599-4

**Published:** 2020-05-11

**Authors:** Kayoko Waki, Kanako Yokomizo, Kouichiro Kawano, Naotake Tsuda, Nobukazu Komatsu, Akira Yamada

**Affiliations:** 1 Cancer Vaccine Development Division, Research Center for Innovative Cancer Therapy, Kurume University, Kurume, Fukuoka 830-0011 Japan; 2 Department of Obstetrics and Gynecology, Kurume University School of Medicine, Kurume, Fukuoka Japan; 3 Department of Immunology, Kurume University School of Medicine, Kurume, Fukuoka Japan

**Keywords:** CTL, DNA integrity, IgG, Ovarian cancer, Peptide vaccine

## Abstract

Cancer immunotherapy including vaccine therapy is a promising modality for cancer treatment, but few patients show its clinical benefits currently. The identification of biomarkers that can identify patients who will benefit from cancer immunotherapy is thus important. Here, we investigated the potential utility of the circulating cell-free DNA (cfDNA) integrity—a ratio of necrotic cell-derived, longer DNA fragments versus apoptotic cell-derived shorter fragments of Alu gene—as a biomarker of vaccine therapy for patients with ovarian cancer. We analyzed plasma samples from 39 patients with advanced or recurrent ovarian cancer enrolled in clinical trials for personalized peptide vaccinations. We observed that (1) the cfDNA integrity was decreased after the first cycle of vaccination, and (2) the decreased levels of cfDNA integrity were correlated with vaccine-induced immune responses; i.e., decreased cfDNA integrity was observed in 91.7% and 59.3% of the IgG-positive and negative patients, respectively (*p* = 0.0445). Similarly, decreased cfDNA integrity was observed in 92.9% and 56.0% of CTL response-positive and negative patients, respectively (*p* = 0.0283). These results suggest that the circulating cfDNA integrity is a possible biomarker for cancer vaccine therapy.

## Introduction

Ovarian cancer is the eighth most common cancer in women, and each year worldwide, nearly 300,000 women newly develop ovarian cancer and 185,000 individuals die from it [1]. Taxane and platinum-based chemotherapy and/or bevacizumab (an anti-angiogenic molecular targeting agent) are widely used to treat ovarian cancer [2]. The presence of tumor-infiltrated T lymphocytes and the expression of cytotoxic T-lymphocyte (CTL)-directed tumor antigens in ovarian cancer tissues have been reported [3–5], which suggests that immunotherapy could become a promising modality for the treatment of ovarian cancer.

We have conducted clinical studies of a peptide-based cancer vaccine in patients with advanced or recurrent ovarian cancer [6]. The cancer vaccine consisted of 31 CTL-epitope peptides, and a maximum of four peptides was selected from among the 31 peptides based on each patient's HLA-A locus types and pre-vaccination immunity to the peptides; we thus refer to it as the "personalized peptide vaccination". Although promising results such as the improvement of overall survival in the vaccine-treated group were obtained, only a limited number of patients received a clinical benefit from the vaccine [6–11]. The development of biomarkers that can be used to identify the patients who will benefit from the personalized peptide vaccination is thus an urgent and important issue.

We have reported several biomarkers related to the prognosis of vaccine-treated patients with various cancers including ovarian cancer [6–18]. The early induction of a vaccine-specific IgG response was correlated with preferable prognosis [6–9, 11]. Inflammation-related biomarkers such as C-reactive protein (CRP), interleukin (IL)-6, serum amyloid A (SAA), CCL-2/MCP-1, and high-mobility group box 1 (HMGB1) were confirmed as biomarkers that are correlated with poor prognosis [12–18]. However, it is not yet possible to precisely identify the patients in a vaccine-treated group who will benefit from the vaccine.

A recent approach to the identification of new biomarkers is the use of a liquid biopsy. Cell-free DNA in the plasma contains tumor cell-derived DNA. The physiological cell death of normal cells is mostly apoptosis, whereas pathologic tumor cell death is mainly necrosis [19]. Apoptotic cell-derived DNA fragments are generally < 200 bp in length, whereas necrotic cell-derived DNA fragments are more random in size and contain longer sizes of DNA (~ 200–400 bp) [20]. The circulating cell-free DNA (cfDNA) integrity, calculated as the ratio of the longer DNA fragment concentration to the shorter DNA fragment concentration, has been investigated in the efforts to find biomarkers for the diagnosis of various cancers [21, 22]. The Alu element, which is the most abundant repetitive element in the human genome, is frequently used in these efforts, and it is the most reliable target of DNA integrity [21, 22]. We conducted the present study to investigate the possibility of using the circulating cfDNA integrity as a biomarker of vaccine therapy for patients with ovarian cancer.

## Patients and Methods

### Plasma samples

Frozen plasma samples from 39 patients with advanced or recurrent ovarian cancer who were enrolled in clinical trials of the personalized peptide vaccination during the period from January 2009 to July 2012 were used in this study [6]. The histology of the ovarian cancers was as follows: serous adenocarcinoma (*n* = 22), endometrioid carcinoma (*n* = 6), mucinous adenocarcinoma (*n* = 3), clear cell carcinoma (*n* = 3), and others (*n* = 5). The patients' stages were as follows: stage IIIa (*n* = 1), stage IIIc (*n* = 6), stage IV (*n* = 2), and recurrent (*n* = 30). The performance status (PS) values were 0 (*n* = 32) and 1 (*n* = 7).

The personalized peptide vaccination clinical protocols have been reported [6]. In brief, each peptide was emulsified with Montanide ISA51VG (SEPPIC, Paris, France) and a maximum of four different peptides was subcutaneously injected. One cycle of the vaccine treatment consisted of a weekly injection for 6 or 8 weeks. Plasma samples were obtained before and after the first and second cycles of vaccination and stored at − 80 °C. The clinical studies had been approved by the Kurume University Ethics Committee and registered with the UMIN Clinical Trial Registry under trial numbers UMIN3083 and UMIN1482.

### Cell-free DNA integrity

For the determination of the cfDNA integrity, the patients' unpurified plasma samples were used as cfDNA for the amplification of Alu fragments. Frozen plasma samples were thawed at room temperature and centrifuged at 16,000 × *g* for 5 min at 4 °C to remove insoluble materials. The supernatants were subsequently diluted 1:40 with distilled water, and 1.5 μL of each sample was subjected to the subsequent polymerase chain reaction (PCR) in a total volume of 15 μL. The amplification and quantitation of short and long Alu fragments were performed using a real-time PCR system (StepOne plus, Thermo Fisher Scientific, Waltham, MA) with THUNDERBIRD SYBR qPCR mix (Toyobo, Osaka, Japan). Short (115-bp) and long (247-bp) PCR fragments of Alu reflect the total cfDNA and tumor cell-derived cfDNA, respectively.

The PCR primer pairs were as follows: forward, 5′-CCTGAGGTCAGGAGTTCGAG-3′ and reverse, 5′-CCTGAGGTCAGGAGTTCGAG-3′ for Alu-115; forward, 5′-GTGGCTCACGCCTGTAATC-3′ and reverse, 5′-CAGGCTGGAGTGCAGTGG-3′ for Alu-247. Amplification was performed 40 cycles at 95 °C for 30 s, 64 °C for 30 s, and 72 °C for 30 s, following the initial denaturation at 95 °C for 10 min. An arbitrary cutoff value of delta △*Rn* = 0.65 was used to obtain Ct values. The cfDNA integrity was calculated according to the formula:

$$ {\text{cfDNA}}\;{\text{integrity}} = {2}^{{({\text{Ct}}\;{\text{value}}\;{\text{of}}\;{\text{Alu}} - {115} - {\text{Ct}}\;{\text{value}}\;{\text{of}}\;{\text{Alu}} - {247})}} $$

### Measurement of peptide-reactive IgG and CTLs

The plasma IgG levels to the vaccine peptides were measured using the Luminex analyzer (Luminex, Austin, TX) as described [6]. If the patients' peptide-specific IgG levels were increased to ≥ twofold of the pre-vaccination level, the response was considered augmented.

The CTL responses were measured by an interferon-gamma (IFN-γ) ELISPOT assay as described [6]. If the spot number was increased to ≥ twofold of the pre-vaccination number, the response was considered augmented.

### Statistical analyses

Changes in the Alu-115, Alu-247, and cfDNA integrity levels between the pre-vaccination and post-vaccination samples were analyzed by paired t test. The cfDNA integrity and immune response rates were compared by t test. The distribution of the cases in which the cfDNA integrity increased or decreased to the different histological types of ovarian cancer was analyzed by Chi-square test. The statistical analyses were performed using JMP Pro ver. 13 software (SAS, Cary, NC).

## Results

### Alteration of the circulating cfDNA integrity during the peptide vaccination

**Fig. 1:**
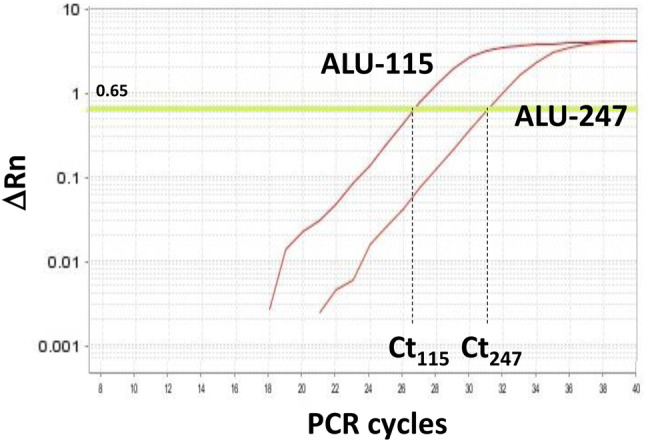
Representative amplification curves for Alu-115 and Alu-247. An arbitrary cutoff value of ∆Rn = 0.65 was used to obtain the Ct values

**Fig. 2:**
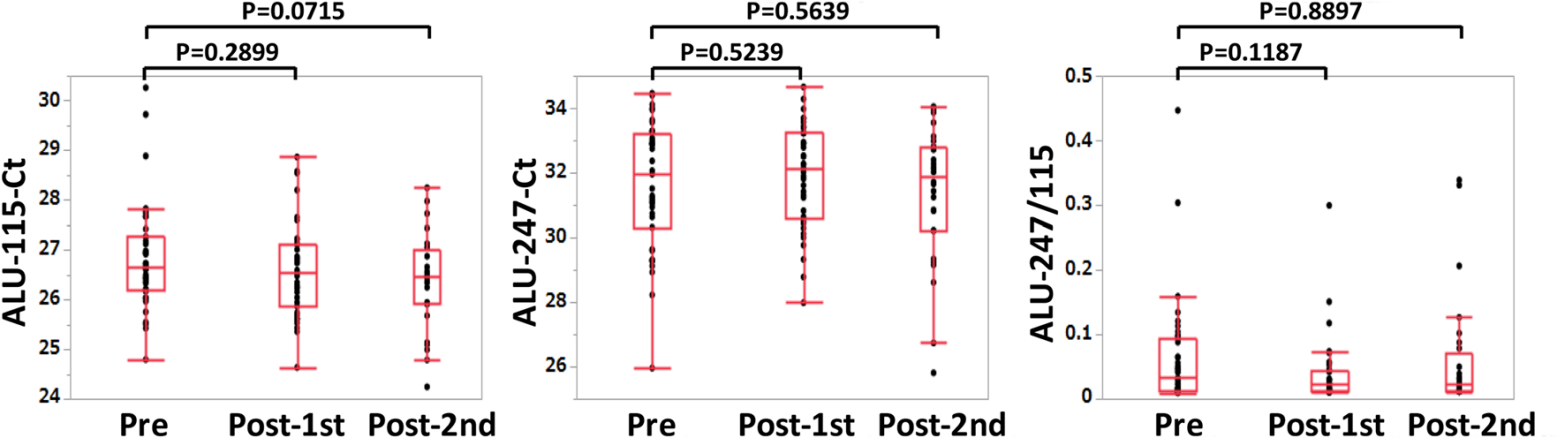
Total cfDNA (Alu-115), tumor cell-derived cfDNA (Alu-247), and the cfDNA integrity (Alu-247/Alu-115) of pre-, post-1 and 2 cycle vaccination are shown

We analyzed the circulating cfDNA integrity of 39 patients with advanced or recurrent ovarian cancer who were treated with a personalized peptide vaccination in a clinical trial [6]. The total cfDNA (Alu-115: the short 115-bp PCR fragment of Alu) and the tumor cell-derived cfDNA (Alu-247: the long 247-bp PCR fragment of Alu) of each sample were quantified by real-time PCR, and the cfDNA integrity was calculated. Representative PCR amplification curves are shown in Fig. 1. The total cfDNA (Alu-115), tumor cell-derived cfDNA (Alu-247), and the cfDNA integrity (Alu-247/Alu-115) of pre- and post-first and second cycles of vaccination are provided in Fig. 2.

**Fig. 3:**
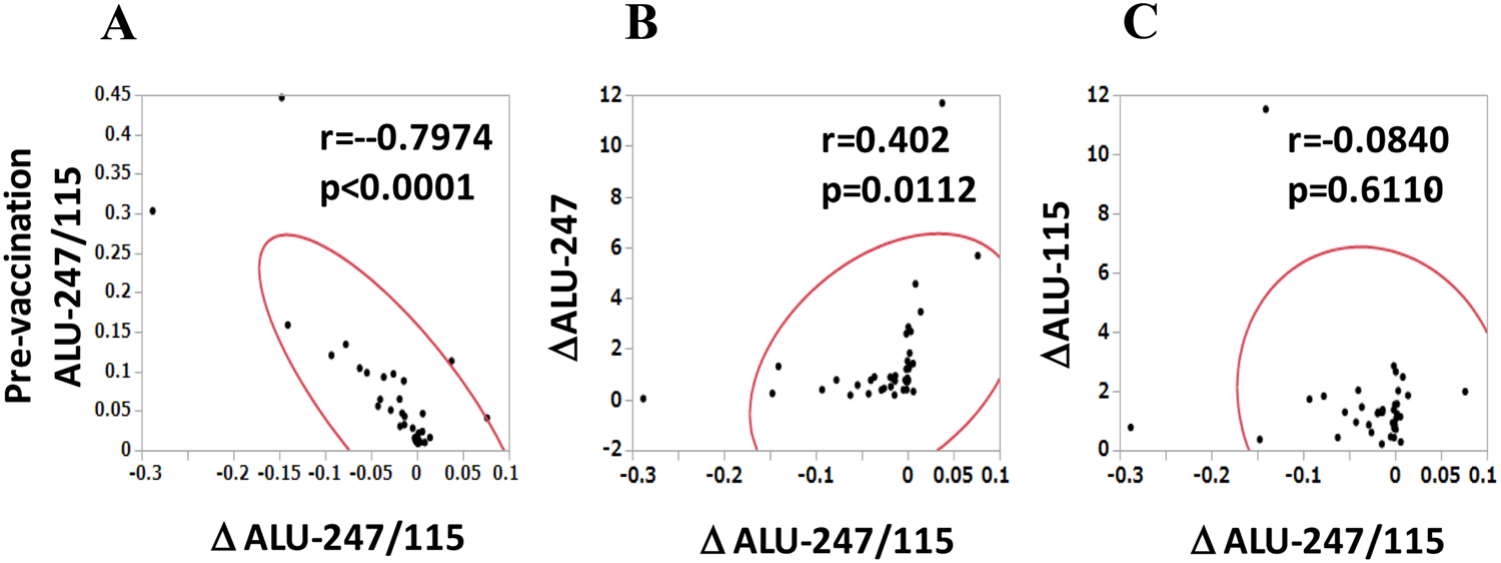
The relationships between the alteration of cfDNA integrity (∆Alu-247/115) during the first cycle of vaccination and the pre-vaccination cfDNA integrity (**a**), between ∆Alu-247/115 and the alteration of Alu-247 (∆Alu-247) during the first cycle of vaccination (**b**), and between ∆Alu-247/115 and the alteration of Alu-115 (∆Alu-115) during the first cycle of vaccination (**c**) are shown

**Fig. 4:**
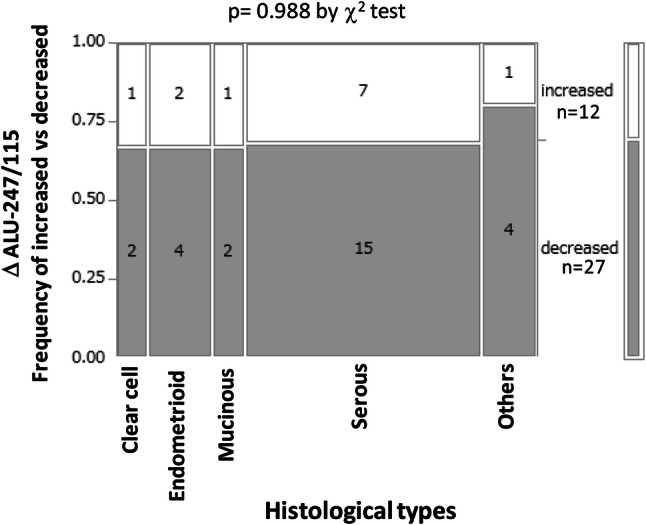
The relationship between the histological types and cfDNA integrity. The distribution of the cases in which the cfDNA integrity increased or decreased to the different histological types of ovarian cancer was analyzed by Chi-square test. Numbers in the columns: the number of patients

Significant alterations in Alu-115 and Alu-247 were not observed during the first cycle of vaccination, whereas the cfDNA integrity was decreased during the vaccination, and the alteration was inversely and significantly correlated with the pre-vaccination levels of the cfDNA integrity (*r* =  − 0.7974, *p* < 0.0001). The alteration of the cfDNA integrity during the first vaccination reflected mostly the changes in Alu-247 (*r* = 0.402, *p* = 0.0112) (Fig. 3). The cases in which the circulating cfDNA integrity increased or decreased were equally distributed to the different histological types of ovarian cancer (*p* = 0.988 by Chi-square test, Fig. 4), and no correlation was observed between cfDNA integrity and histological types. It should be noted that no relationship was observed between each of these parameters, including histological types, and the overall survival of the patients (data not shown).

### The relationship between the circulating cfDNA integrity and the vaccine-induced IgG response

**Fig. 5:**
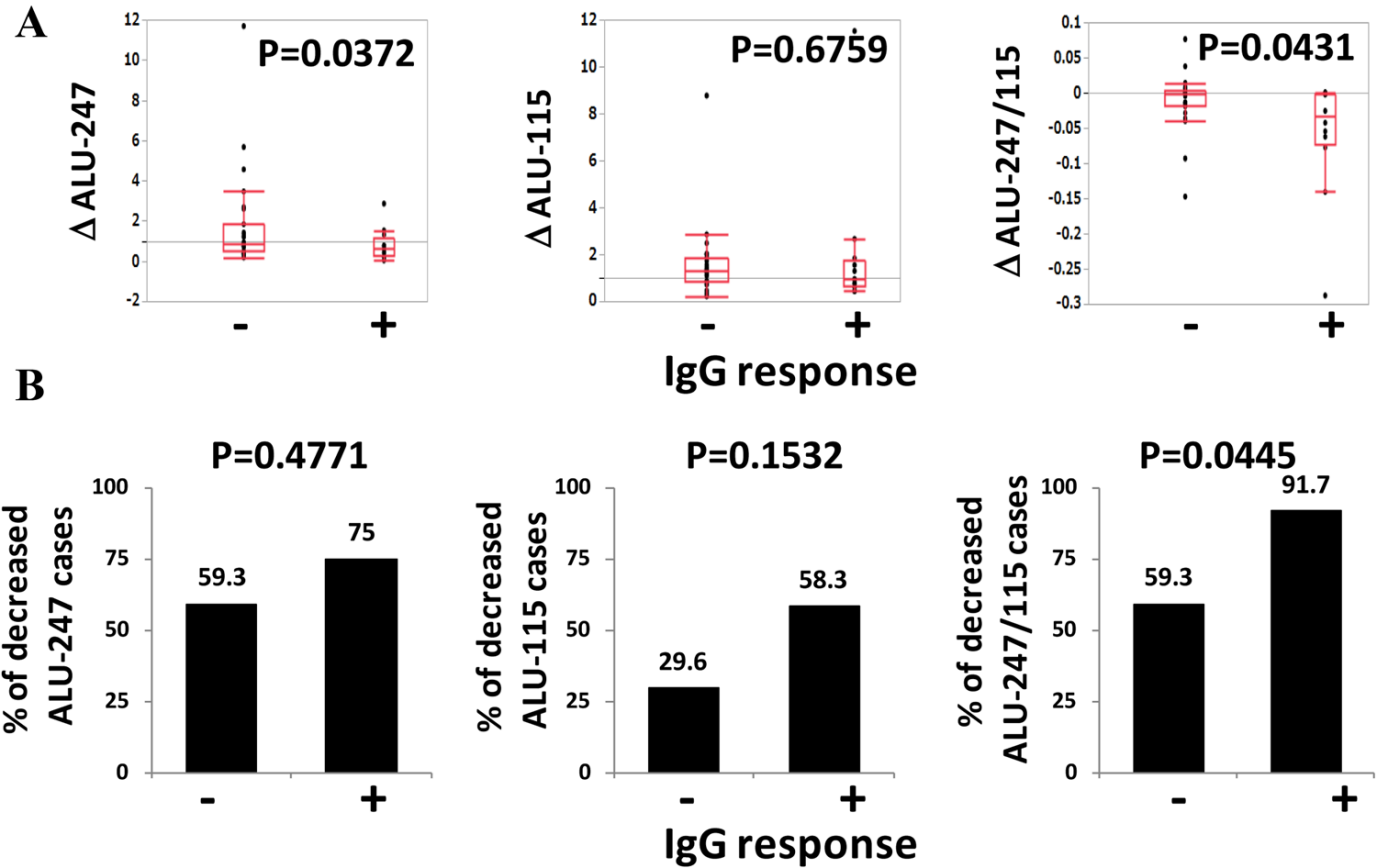
The relationship between the circulating cfDNA integrity and the vaccine-induced IgG response. **a** The alterations of Alu-247 (∆Alu-247), Alu-115 (∆Alu-115), and the cfDNA integrity (∆Alu-247/115) during the first cycle of vaccination are plotted for the IgG responder and IgG non-responder groups. **b** The percentages of decreased Alu-247 cases, decreased Alu-115 cases, and decreased cfDNA integrity cases in the IgG responder and IgG non-responder groups are shown

We also analyzed the relationship between the decreased cfDNA integrity observed after the first cycle of vaccination and the vaccine-induced IgG response. We observed an augmentation of IgG against the vaccinated peptides after the first cycle of vaccination in 12 of the 39 patients. The alterations of Alu-115, Alu-247, and the cfDNA integrity after the first cycle of vaccination in the IgG response-positive (= augmented) and negative groups are shown in Fig. 5. The decrease in the cfDNA integrity of the IgG response-positive group (*n* = 12) was significantly larger than that of the IgG response-negative group (*n* = 27) (*p* = 0.043) (Fig. 5a). Decreased cfDNA integrity was observed in 91.7% of the IgG-positive group but in only 59.3% of the IgG-negative group (*p* = 0.044) (Fig. 5b). A similar tendency was observed in the alteration of Alu-247 (*p* = 0.037) but not in that of Alu-115 (Fig. 5a). The relationship between cfDNA integrity and vaccine-induced IgG responses after the second or later cycle of vaccination was not analyzed, since most of the patients (96%) became IgG positive after the second cycle of vaccination. Analyses of the CTL response after the second or later cycle of vaccination were not performed due to the insufficient number of PBMC samples.

### The relationship between the circulating cfDNA integrity and the vaccine-induced CTL response

**Fig. 6:**
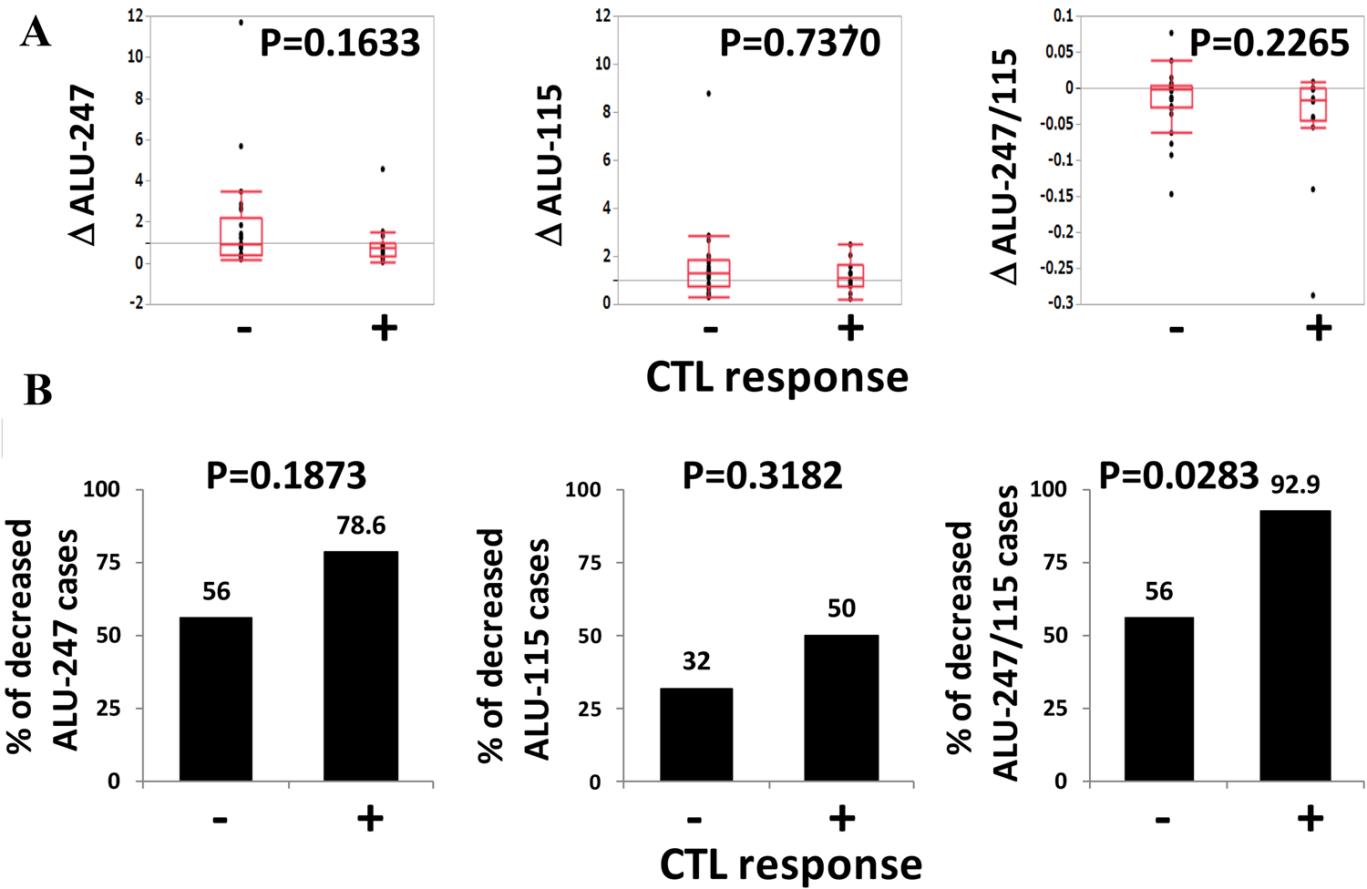
The relationship between the circulating cfDNA integrity and the vaccine-induced CTL response. **a** The alterations of Alu-247 (∆Alu-247), Alu-115 (∆Alu-115), and cfDNA integrity (∆Alu-247/115) during the first cycle of vaccination are plotted in the CTL responder and CTL non-responder groups. **b** The percentages of decreased Alu-247 cases, decreased Alu-115 cases, and decreased cfDNA integrity cases in the CTL responder and CTL non-responder groups are shown

We next analyzed the relationship between the decreased cfDNA integrity observed after the first cycle of vaccination and the vaccine-induced CTL response (Fig. 6). An augmentation of the CTL response against the vaccinated peptides after the first cycle of vaccination was observed in 14 of the 39 patients. No significant alteration in Alu-115, Alu-247, or the cfDNA integrity after the first cycle of vaccination was observed in the CTL response-positive (*n* = 14) or negative (*n* = 25) groups (Fig. 6a). However, decreased cfDNA integrity was observed in 92.9% of the CTL response-positive group and 56.0% of the CTL response-negative group (*p* = 0.0283) (Fig. 6b).

## Discussion

We observed that the cfDNA integrity was decreased at the end of the first cycle of vaccination, and we analyzed the relationship between the decreased cfDNA integrity and vaccine-induced immune responses. The decrease in cfDNA integrity in the IgG response-positive patients was significantly greater than that in the IgG response-negative patients, and although nearly 92% of the IgG-positive patients showed decreased cfDNA integrity, significantly fewer IgG-negative patients showed it (~ 60%). Similarly, nearly 93% of the patients who had a positive CTL response showed decreased cfDNA integrity, whereas only 56% of the patients with a negative response did. These data demonstrate a clear correlation between decreased cfDNA integrity and vaccine-induced immune responses.

Immune cell (e.g., CTL and natural killer cell)-mediated tumor cell death is usually apoptosis, not necrosis, and DNA fragmentation occurs in apoptotic cells. It is thus conceivable that the decrease in cfDNA integrity that we observed after the first vaccination was due to an increase of Alu-115. However, the decrease in cfDNA integrity reflected mainly the decrease of Alu-247. One possible reason for this is that a partial conversion of the tumor cells' death from necrosis to apoptosis induced a reduction of Alu-247. The effect of this conversion was less on the Alu-115 levels since Alu-115 reflects the total plasma cell-free DNA (including both apoptosis- and necrosis-derived DNA).

There are many studies regarding cfDNA integrity as a biomarker of various cancers for diagnosis and prognosis [21–23]. In their study of ovarian cancer, Zhang et al. reported that plasma levels of patients with a long Alu fragment (Alu-219) and cfDNA integrity were significantly higher than those of the benign-disease and healthy control groups [23].

To the best of our knowledge, there is only one published report regarding the plasma DNA integrity of patients treated with a cancer vaccine. Kitahara et al. [24] analyzed the pre-vaccination levels of the plasma cfDNA integrity of patients with metastatic colorectal cancer treated with a combination of a cancer vaccine and chemotherapy as the first-line therapy, and they reported that the progression-free survival of the patients with low levels of plasma cfDNA integrity was significantly longer than that of the high cfDNA integrity group [24]. In that report, only the pre-vaccination levels of cfDNA integrity are discussed; there is no information regarding post-vaccination values. Our present investigation thus provides the first report regarding alterations of circulating cfDNA integrity during a peptide vaccination.

In conclusion, our analyses demonstrated that (1) the cfDNA integrity was decreased after the first cycle of vaccination in patients with advanced ovarian cancer, and (2) decreased levels of cfDNA integrity were correlated with vaccine-induced immune responses. These results suggest that the circulating cfDNA integrity is a possible biomarker for cancer vaccine therapy.
